# Rapid divergence and diversification of mammalian duplicate gene functions

**DOI:** 10.1186/s12862-015-0426-x

**Published:** 2015-07-15

**Authors:** Raquel Assis, Doris Bachtrog

**Affiliations:** Department of Biology, Huck Institutes of Life Sciences, Center for Medical Genomics, Pennsylvania State University, University Park, PA 16802 USA; Department of Integrative Biology, Center for Theoretical Evolutionary Genomics, University of California, Berkeley, CA 94720 USA

**Keywords:** Gene duplication, Duplicate genes, Neofunctionalization, Subfunctionalization, Specialization

## Abstract

**Background:**

Gene duplication provides raw material for the evolution of functional innovation. We recently developed a phylogenetic method that classifies evolutionary processes driving the retention of duplicate genes by quantifying divergence between their spatial gene expression profiles and that of their single-copy orthologous gene in a closely related sister species.

**Results:**

Here, we apply our classification method to pairs of duplicate genes in eight mammalian genomes, using data from 11 tissues to construct spatial gene expression profiles. We find that young mammalian duplicates are often functionally conserved, and that expression divergence rapidly increases over evolutionary time. Moreover, expression divergence results in increased tissue specificity, with an overrepresentation of expression in male kidney, underrepresentation of expression in female liver, and strong underrepresentation of expression in testis. Thus, duplicate genes acquire a diversity of new tissue-specific functions outside of the testis, possibly contributing to the origin of a multitude of complex phenotypes during mammalian evolution.

**Conclusions:**

Our findings reveal that mammalian duplicate genes are initially functionally conserved, and then undergo rapid functional divergence over evolutionary time, acquiring diverse tissue-specific biological roles. These observations are in stark contrast to the much faster expression divergence and acquisition of broad housekeeping roles we previously observed in *Drosophila* duplicate genes. Due to the smaller effective population sizes of mammals relative to *Drosophila*, these analyses implicate natural selection in the functional evolution of duplicate genes.

**Electronic supplementary material:**

The online version of this article (doi:10.1186/s12862-015-0426-x) contains supplementary material, which is available to authorized users.

## Background

Gene duplication produces copies of existing genes, which can diverge from their ancestral states and contribute to the evolution of novel phenotypes. A large proportion of mammalian genes arose via gene duplication [1, 2], many of which are members of large gene families with diverse and important functions. For example, Hox, opsin, and olfactory receptor gene families were all produced by gene duplication [3, 4]. However, the evolutionary paths leading from redundant copies to distinct genes with essential functions remain unclear.

Different processes may drive the long-term retention of duplicate genes: Parent and child copies may each maintain the function of their single-copy ancestral gene (conservation [5]); one copy may maintain the ancestral function, while the other acquires a new function (neofunctionalization [5]); each copy may lose part of its function, such that together both copies carry out the ancestral function (subfunctionalization [6–8]); or both copies may acquire new functions (specialization, also called subneofunctionalization or neosubfunctionalization [9]). We recently developed a phylogenetic method that utilizes distances between gene expression profiles to classify these evolutionary processes (see [10] and Methods for details). Our method can be applied to pairs of duplicates and requires that, for each pair, we can distinguish between parent and child copies and identify their single-copy ortholog (referred to as “outgroup gene” here, and as “ancestral gene” in [10]) in a closely related sister species. Moreover, parent, child, and outgroup genes must all have spatial or temporal gene expression data from which expression profiles can be constructed.

To study the roles of conservation, neofunctionalization, subfunctionalization, and specialization in the retention of mammalian duplicate genes, we applied our method to pairs of duplicate genes in eight mammalian genomes: human (*Homo sapiens*), chimpanzee (*Pan trogodytes*), gorilla (*Gorilla gorilla*), orangutan (*Pongo pygmaeus abelii*), macaque (*Macaca mulatta*), mouse (*Mus musculus*), opossum (*Monodelphis domestica*), and platypus (*Ornithorhynchus anatinus*). Using synteny information from whole-genome alignments to determine orthologous genomic positions, and parsimony to infer gene acquisitions, we distinguished between parent and child copies and identified single-copy outgroup genes for each pair of duplicates (see Methods for details). Then, we applied our classification method to RNA-seq data from 11 mammalian tissues: female and male cerebrum, female and male cerebellum, female and male heart, female and male kidney, female and male liver, and testis [11].

## Results

We obtained 654 pairs of mammalian duplicate genes for which we could distinguish between parent and child copies and also identify at least one expressed single-copy outgroup gene in a closely related sister species. Application of our method to these pairs yielded 382 cases of conservation, 213 cases of neofunctionalization (105 neofunctionalized parent copies and 108 neofunctionalized child copies), 9 cases of subfunctionalization, and 50 cases of specialization (Additional file 1: Table S1; see Methods for details). Thus, most mammalian duplicate genes have conserved expression profiles. Moreover, expression divergence is often asymmetric between duplicates, and retention of duplicates by subfunctionalization is rare.

**Fig. 1:**
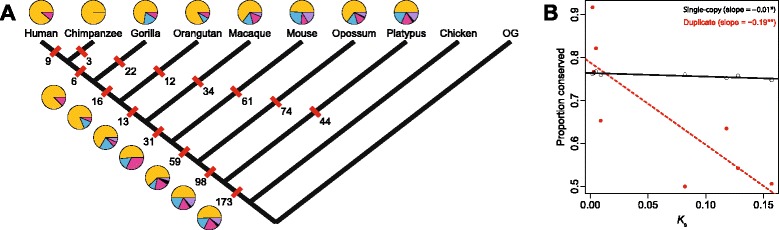
Evolutionary processes driving the retention of mammalian duplicate genes. **a** Pie charts depicting the role of each process on different branches of the mammalian phylogeny (yellow = conservation; blue = neofunctionalization of parent copy; pink = neofunctionalization of child copy; black = subfunctionalization; purple = specialization). Numbers of duplicate gene pairs examined along specific branches are indicated beside red tick marks. Additional outgroups (OG) used to date duplicates were lizard (*Anolis carolinensis*) and fugu (*Takifugu rubripes*). **b** Relationship of median *K*_s_ between pairs of species (human-chimpanzee, human-gorilla, human-orangutan, human-macaque, human-mouse, human-opossum, human-platypus, and human-chicken) to proportions of functionally conserved single-copy (black) and duplicate (red) genes. Least-squares linear regression lines and their slopes are depicted to show rates of decreased functional conservation in single-copy (black) and duplicate (red) genes. *p < 0.05; **p < 0.01; *p* < 0.001 (see Methods for details)

The availability of data from species of different evolutionary distances along the mammalian phylogeny enabled us to investigate whether expression divergence increases with the age of duplicate genes, as expected if genes evolve new functions over time. We used parsimony to date acquisitions of child copies along the mammalian phylogeny (Fig. 1a; see Methods for details). Consistent with global patterns, conservation is the most common evolutionary process underlying the retention of duplicate genes in every mammalian lineage surveyed (Fig. 1a). To examine how functional conservation changes over time, we compared proportions of functionally conserved duplicates between pairs of sister species separated by varying evolutionary distances (Fig. 1b). We estimated the evolutionary distance between each pair of sister species by calculating the median synonymous sequence divergence rate (*K*_s_) between all single-copy genes in the two species, though using the median nonsynonymous sequence divergence rate (*K*_a_) produced similar patterns (see Additional file 2: Figure S1). Indeed, this analysis revealed that the proportion of duplicate genes with conserved expression profiles decreases with evolutionary distance between species (Fig. 1b), suggesting that young mammalian duplicates are generally functionally conserved, and that new functions evolve over time. Moreover, while the proportion of functionally conserved single-copy genes also decreases with evolutionary distance between species, the magnitude of the slope of the least-squares linear regression line for single-copy genes is an order of magnitude smaller than for duplicate genes (Fig. 1b). Thus, expression divergence of duplicate genes occurs rapidly in mammals.

**Fig. 2:**
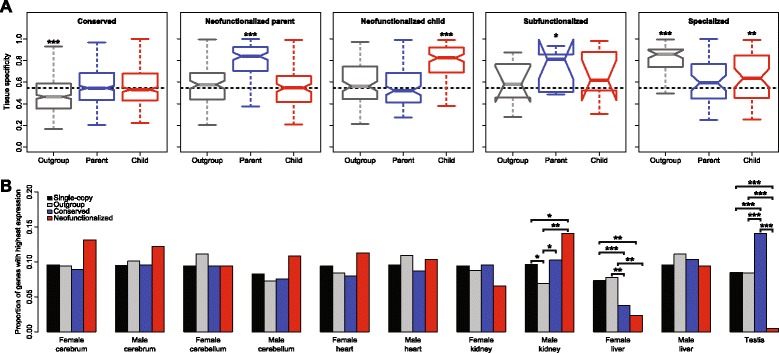
Comparison of tissue-specific expression among outgroup, parent, and child genes in different classes. **a** Boxplots showing distributions of tissue specificities for outgroup (gray), parent (blue), and child (red) genes in each class. Dotted black lines represents the median tissue specificity for single-copy genes, and asterisks show significance relative to the distribution for single-copy genes. **b** Barplots depicting proportions of single-copy (black), outgroup (gray), functionally conserved (blue) and neofunctionalized (red) genes with highest expression in each tissue. Absolute counts for each bar are provided in Additional file 1: Table S3. Asterisks above lines connecting two bars indicate significance between groups. *p < 0.05; **p < 0.01; *p* < 0.001 (see Methods for details)

We next wanted to investigate the types of novel functions acquired by mammalian duplicate genes. To address this question, we first compared tissue specificities of outgroup, parent, and child genes in each class to those of single-copy genes, which represent typical genes that have not changed in copy number (Fig. 2a). We used the highest relative expression level for each gene as a measure of its tissue specificity. In the conserved class, outgroup genes tend to be more broadly expressed than single-copy genes, whereas parent and child copies have typical tissue specificities. In both neofunctionalized classes (parent and child), outgroup genes and duplicate gene copies with conserved expression profiles have typical tissue specificities, whereas gene copies with diverged expression profiles are highly tissue-specific. Because the sample size of the subfunctionalized class is small, we must be cautious in making generalizations. However, based on the current sample, it appears that outgroup and child genes have typical tissue specificities, whereas parent copies have increased tissue specificities. In the specialized class, outgroup genes are highly tissue-specific, while both parent and child genes are more broadly expressed, with child genes displaying slightly elevated tissue specificities relative to typical genes. These patterns suggest that functional divergence of both duplicates may occur when the ancestral gene is tissue-specific, resulting in broadening of expression patterns in parent and child copies. In contrast, asymmetric acquisition of a new function by neofunctionalization may occur when the ancestral gene is broadly expressed, resulting in one gene copy becoming highly tissue-specific.

To determine the types of tissue-specific functions that arise under neofunctionalization, we compared proportions of single-copy, outgroup, functionally conserved (from conserved and neofunctionalized classes), and neofunctionalized genes with highest expression levels in each tissue (Fig. 2b; Additional file 1: Table S2). We observed significant differences in male kidney, female liver, and testis tissues. Relative to single-copy genes, there was an underrepresentation of outgroup genes and an overrepresentation of neofunctionalizad genes with highest expression in male kidney. Additionally, relative to outgroup genes, there were overrepresentations of conserved and neofunctionalized genes with highest expression in male kidney. These patterns suggest that ancestral genes are deficient in male kidney expression, which generally increases in both gene copies after duplication. Also, relative to both single-copy and outgroup genes, there were underrepresentations of conserved and neofunctionalized genes with highest expression in female liver tissue. This is suggestive of a general decrease in female liver tissue expression in both gene copies after duplication. Finally, relative to both single-copy and outgroup genes, there was an overrepresentation of conserved and a severe underrepresentation (only one gene) of neofunctionalized genes with highest expression in testis. This indicates that after duplication, testis expression increases in conserved copies and decreases in neofunctionalized copies. Thus, unlike the trends observed in male kidney and female liver, both copies alter their testis expression in opposite ways, such that tissue-specific neofunctionalized copies are highly underrepresented in testis.

## Discussion

Studies of duplicate genes have shown that expression divergence between copies occurs rapidly [12–21] and is often asymmetric [13, 16, 19, 20]. Moreover, differences between expression levels of single-copy and duplicate genes and their relationships to neofunctionalization and subfunctionalization have also been studied previously [22, 23]. However, our analysis is the first to utilize gene expression data and phylogenetic relationships among species to classify the evolutionary processes driving the retention of mammalian duplicates on a genome-wide scale.

In a previous study, we applied our classification method to duplicate genes in *Drosophila melanogaster* and *D. pseudoobscura* [10]. However, in our *Drosophila* dataset, *K*_s_ ranged from 0.11 (between *D. melanogaster* and *D. simulans* [24]) to 1.79 (between *D. melanogaster* and *D. pseudoobscura* [25]). In our mammalian dataset, *K*_s_ ranges from 0.01 (between human and chimpanzee [26]) to 1.41 (between human and platypus [27]). Thus, the smallest *K*_s_ in our mammalian dataset is an order of magnitude smaller than in our *Drosophila* dataset, enabling us to capture much younger duplicates in our current analysis. Moreover, our current dataset contains gene expression profiles from nine vertebrate species at varying evolutionary distances, compared to only three species in *Drosophila*. This provided us with greater temporal resolution in mammals than in *Drosophila*, and allowed us to more closely examine the functional diversification of mammalian duplicates over evolutionary time.

Contrary to our observation in mammalian duplicates, we found that most *Drosophila* duplicates were neofunctionalized, and examination of evolutionary processes over shorter divergence times suggested that novel functions arise within a few million years of evolution [10]. This difference may be due to the larger effective population size (*N*_e_) of *Drosophila* than of mammals [28–30], which contributes to more efficient adaptive protein and regulatory sequence evolution in *Drosophila* [31–33], and could similarly result in more rapid acquisition of adaptive functions by *Drosophila* duplicate genes. Even so, expression divergence of duplicate genes occurs much faster than that of single-copy genes in mammals. Thus, though natural selection may not be as efficient as in *Drosophila*, it still appears to play an important role in the functional divergence of duplicate genes in mammals.

While small *N*_e_ is also thought to result in a higher prevalence of subfunctionalization [34], this process does not appear to play a major role in the retention of duplicate genes in either lineage. One possible reason for this observation is that subfunctionalization may be more common in duplicate genes produced by whole genome duplication events [18, 35], which our study does not examine. Another possibility is that the stringency of our subfunctionalization classification resulted in an underestimation of such cases. Because our cutoff for expression divergence was conservative (see Methods), this would have most likely resulted in subfunctionalized genes being grouped with conserved genes. However, decreasing the cutoff increases the number of specialized, rather than subfunctionalized, genes (Additional file 1: Table S3). One potential solution to this problem is to apply our method to a dataset consisting of more tissues, which may help better differentiate functions of genes, resulting in the classification of fewer conserved duplicates.

Another difference between our findings in *Drosophila* and mammals was that neofunctionalization primarily occurred in child copies in *Drosophila* [10], whereas it occurred with equal frequency in child and parent copies in mammals. This may also be attributed to differences in efficiencies of natural selection between *Drosophila* and mammals. Under neutrality, most duplicate genes should be lost within the first few million years of evolution [36]. In *Drosophila*, many neofunctionalized child genes likely arose with or quickly acquired new beneficial functions that were retained by natural selection [10]. In mammals, for which natural selection is less efficient, such genes may be lost more often. However, new genes with conserved functions may be more easily maintained. In particular, recent studies of mammalian duplicate genes have shown that transcription of one duplicate is often suppressed by methylation, and that methylation decreases over evolutionary time [37, 38]. Thus, child copies with conserved functions may initially be silenced in mammals. Then, once fixed via a neutral or nearly neutral process, they can be demethylated, enabling them to acquire new functions. Under this scenario, neofunctionalization is likely equally probable in either duplicate, resulting in the relatively similar frequencies of neofunctionalized parent and child copies that we observed.

In both *Drosophila* and mammals, neofunctionalized genes have tissue-specific functions. However, neofunctionalized *Drosophila* genes are primarily testis-specific [10], whereas neofunctionalized mammalian genes are mostly excluded from testis and expressed in a diversity of other tissues. Moreover, in *Drosophila*, comparison of young and old duplicates supported the “out of the testis” hypothesis of new gene emergence, in which new genes arise with testis-specific functions and evolve broader functions over time [39]. According to this hypothesis, testis may facilitate the initial transcription of young genes, while sheltering them from pseudogenization as they acquire new functions [39], making testis an ideal tissue for young genes. In mammals, neofunctionalization happens more slowly, and most neofunctionalized genes are relatively old. Because young mammalian duplicates are often conserved, we can perhaps better understand the initial forces retaining duplicates by examining expression profiles of conserved duplicates. Among conserved duplicates, there is an overrepresentation of highest testis-expressed genes. Thus, this finding may support a special case of the “out of the testis” hypothesis in mammals, in which young genes often acquire higher, but not necessarily specific, expression in testis. Then, as they age, they acquire diverse tissue-specific functions outside of the testis, possibly facilitating the evolution of a multitude of complex phenotypes across species.

## Conclusions

While gene duplication has long been hypothesized to play an important role in the evolution of novel phenotypes, the processes driving the retention of mammalian duplicate genes remained unclear. In this study, we utilized our previously developed classification method to identify the roles of different evolutionary processes in the retention of mammalian duplicate genes. We found that most mammalian duplicate genes are functionally conserved, and that they diverge rapidly over evolutionary time, acquiring a diversity of tissue-specific functions. In contrast, our previous study in *Drosophila* revealed that duplicate genes are primarily retained via neofunctionalization, and that they diverge even faster than in mammals, acquiring broad housekeeping functions. Thus, our current study highlights key differences in the retention of duplicate genes between mammals and *Drosophila* and, moreover, supports the hypothesis that positive selection drives the functional evolution of duplicate genes in both lineages.

## Methods

### Identification of duplicate and single-copy genes

We downloaded protein sequences and annotation files for eight mammals (*Homo sapiens*, *Pan trogodytes*, *Gorilla gorilla*, *Pongo pygmaeus abelii*, *Macaca mulatta*, *Mus musculus, Monodelphis domestica*, and *Ornithorhynchus anatinus*) and three outgroups (*Gallus gallus*, *Anolis carolinensis*, and *Takifugu rubripes*) from the Ensembl database (release 74) at http://www.ensembl.org. We obtained lists of duplicate genes in each mammalian genome from the Ensembl database (release 74) at http://www.ensembl.org, from the Duplicated Genes Database (DGD) at http://www.dgd.genouest.org, and from protein BLAST searches [40], which we performed as previously described [10]. Any annotated genes not on these lists were considered to be single-copy genes, and gene families with more than two copies were excluded from our analysis.

### Phylogenetic dating and identification of outgroup genes

We downloaded whole-genome alignments from Ensembl (http://www.ensembl.org) and UCSC Genome Bioinformatics (http://www.genome.ucsc.edu) databases and extracted syntenic regions in all genomes for each duplicate gene. We used parsimony to phylogenetically date the origin of each pair of duplicates. In particular, we inferred a duplication event that occurred after the divergence of two sister species if one sister contains two gene copies, while the other sister and all outgroups (including non-mammals) contain a single-copy gene. Duplicates that are present in all species or that could not be resolved via parsimony (e.g., tandem duplicates) were removed from our analysis. For each pair, the gene copy aligned to outgroup genes in the whole-genome alignment was designated as the parent, and the copy that did not align to any regions of the outgroup genomes was considered the child. Because annotation of exons may be unreliable in many of the species used, we did not distinguish between DNA- and RNA-mediated duplication mechanisms. Orthologs for single-copy genes were also obtained via synteny and aligned with MACSE [41]. PAML [42] was used to estimate *K*_a_ and *K*_s_ between orthologous pairs of single-copy genes.

### Identification of evolutionary processes maintaining duplicate genes

We quantile-normalized RNA-seq data from mammalian and chicken tissues [11], and restricted our analysis to pairs for which both copies, and one or more single-copy outgroup genes, are expressed (FPKM ≥ 1) in at least one tissue. The expression profile of the single-copy outgroup gene in the most closely related species with available expression data (see Additional file 1: Table S1) was used as a proxy for the expression of the single-copy ancestral gene prior to duplication. We converted all absolute tissue expression levels to their relative expression levels (proportions of total expression), which were used as gene expression profiles for comparison.

Next, we classified the processes retaining pairs of mammalian duplicate genes by applying our previously developed phylogenetic method [10] to expression profiles of parent, child, and outgroup genes. To summarize, we first calculated Euclidian distances between expression profiles of parent and outgroup copies (*E*_*P,O*_), between expression profiles of child and outgroup copies (*E*_*C,O*_), and between the combined parent–child expression profile and that of the outgroup copy (*E*_*P+C,O*_). We next established baseline divergence levels for genes by calculating Euclidian distances between expression profiles of single-copy genes present in both sister species (*E*_S1,S2_), and used these distances to set cutoffs for expression divergence in each pair of species (see Choice of cutoff for expression divergence). Last, we classified each pair of duplicates as conserved, neofunctionalized, subfunctionalized, or specialized by applying previously described rules [10]. In particular, we expect *E*_P,O_ ≤ *E*_S1,S2_ and *E*_C,O_ ≤ *E*_S1,S2_ when duplicates are functionally conserved, *E*_P,O_ > *E*_S1,S2_ and *E*_C,O_ ≤ *E*_S1,S2_ when the parent copy is neofunctionalized, *E*_P,O_ ≤ *E*_S1,S2_ and *E*_C,O_ > *E*_S1,S2_ when the child copy is neofunctionalized, *E*_P,O_ > *E*_S1,S2_, *E*_C,O_ > *E*_S1,S2_, and *E*_P+C,O_ ≤ *E*_S1,S2_ when duplicates are subfunctionalized, and *E*_P,O_ > *E*_S1,S2_, *E*_C,O_ > *E*_S1,S2_, and *E*_P+C,O_ > *E*_S1,S2_ when duplicates are specialized.

### Choice of cutoff for expression divergence

We explored several cutoff values for defining expression divergence (Additional file 1: Table S3). Modifying the cutoff changed numbers of pairs in different classes in predictable ways. In particular, more stringent cutoff values resulted in more pairs classified as conserved, while less stringent values resulted in fewer pairs classified as conserved. However, the main finding was unaffected by the cutoff value. For all cutoffs tested, most duplicates were classified as conserved, and the relative numbers of pairs in parent and child neofunctionalization classes were similar. Of the cutoffs examined, we chose to use the semi-interquartile range from the median because it is robust to outliers, as we did in a previous study of *Drosophila* duplicate genes. In *Drosophila* duplicate genes, the distribution of *E*_S1,S2_ was right-skewed. In the current study, there are 36 distributions to consider—one for each pair of species compared. While most are approximately normally distributed, we wanted to be able to use the same type of cutoff for all comparisons, and so we did not want to use a cutoff that would be sensitive to differences among shapes of distributions. Moreover, we wanted to ensure that our identification of genes with divergent expression profiles was conservative, which appears to be the case when we use the semi-interquartile range from the median as our cutoff.

A final point about cutoff values is that they are expected to increase as a function of evolutionary distance between the species being compared. However, this is not always the case in the present study (Additional file 1: Table S4) and, in fact, cutoff values do not change much in general. One possibility is that this effect is caused by the use of relative, rather than absolute, expression values in calculating distances. While relative values reduce the effects of experimental differences among data for different species [43], they may also reduce true differences among expression profiles to some degree. Thus, the classification approach may be more conservative as a result of this transformation.

### Statistical analyses

Fits of least-squares linear regression lines were tested with *F*-statistics, and all were significant (*p* < 0.05). Two-sided *t*-tests were used to assess significance of slopes shown in Figs. 1b and Additional file 2: Figure S1. Two-sided Mann–Whitney *U* tests were used to compare distributions of tissue specificities of outgroup, parent, and child genes to those of single-copy genes shown in Fig. 2a. Fisher’s Exact tests were used to compare numbers of genes with highest relative expression levels in each tissue among all pairs of groups shown in Fig. 2b (absolute counts provided in Additional file 1: Table S2). Bonferroni corrections were applied to tests involving multiple comparisons. All statistical analyses were performed in the R software environment [44].

### Availability of supporting data

The data sets supporting the results of this article are included within the article and its additional files.

## Additional files

Additional file 1: Table S1. Classifications of evolutionary processes retaining mammalian duplicate genes. **Table S2.** Absolute gene counts for Fig. 2b. **Table S3.** Classifications resulting from application of different cutoffs for expression divergence. **Table S4.** Cutoffs used to determine expression divergence between sister species. Additional file 2: Figure S1. Functional conservation of single-copy and duplicate genes between pairs of species separated by varying evolutionary distances. Relationship of median *K*_a_ between pairs of species (humanchimpanzee, human-gorilla, human-orangutan, human-macaque, human-mouse, human-opossum, human-platypus, and human-chicken) to proportions of functionally conserved single-copy (black) and duplicate (red) genes. Least-squares linear regression lines and their slopes are depicted to show rates of decreased functional conservation in single-copy (black) and duplicate (red) genes. **p* < 0.05; ***p* < 0.01; *p* < 0.001 (see Methods for details).
