# Supplementary material for: Rapid divergence and diversification of mammalian duplicate gene functions
Source: BMC Evol Biol. 2015 Jul 15;15:138. doi: 10.1186/s12862-015-0426-x (PMC4502564; doi:10.1186/s12862-015-0426-x)
Supplement: Additional file 2: Figure S1. — Functional conservation of single-copy and duplicate genes between pairs of species separated by varying evolutionary distances. Relationship of median K a between pairs of species (humanchimpanzee, human-gorilla, human-orangutan, human-macaque, human-mouse, human-opossum, human-platypus, and human-chicken) to proportions of functionally conserved single-copy (black) and duplicate (red) genes. Least-squares linear regression lines and their slopes are depicted to show rates of decreased functional conservation in single-copy (black) and duplicate (red) genes. *p < 0.05; **p < 0.01; p < 0.001 (see Methods for details). [file 12862_2015_426_MOESM2_ESM.pdf]

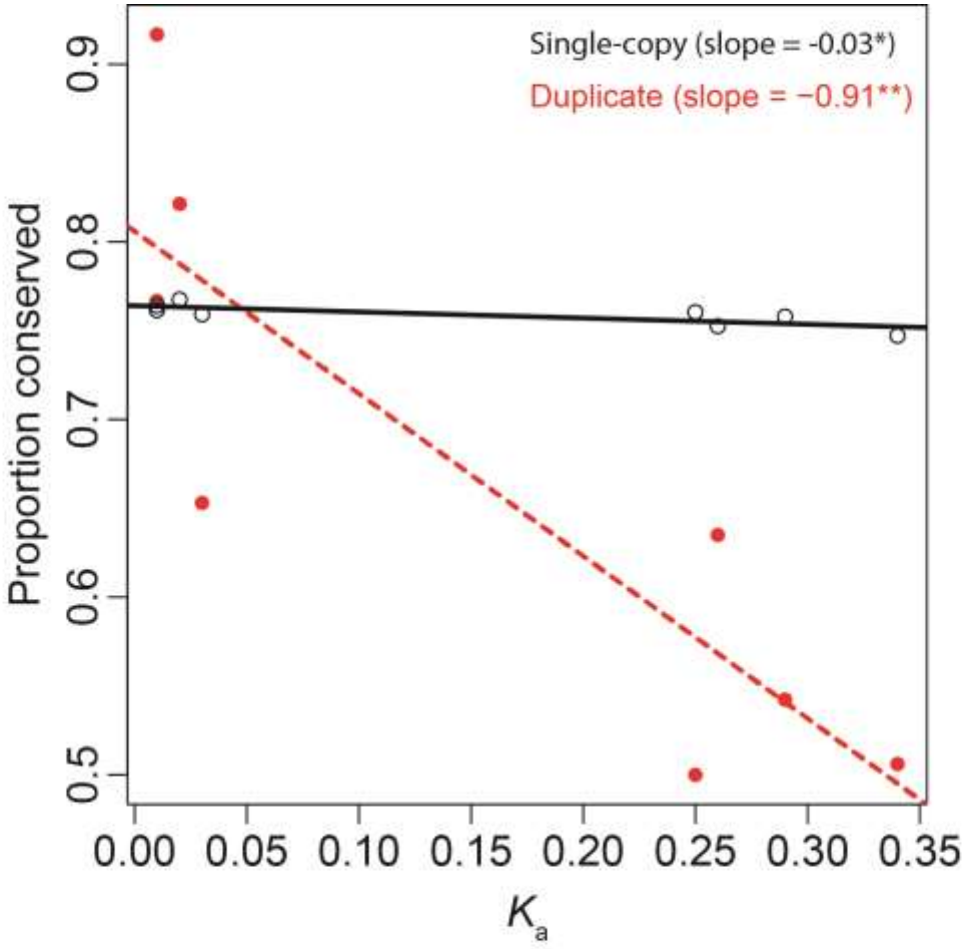

Figure S1. Functional conservation of single-copy and duplicate genes between pairs of species separated by varying evolutionary distances. Relationship of median K_a between pairs of species (human-chimpanzee, human-gorilla, human-orangutan, human-macaque, human-mouse, human-opossum, human-platypus, and human-chicken) to proportions of functionally conserved single-copy (black) and duplicate (red) genes. Least-squares linear regression lines and their slopes are depicted to show rates of decreased functional conservation in single-copy (black) and duplicate (red) genes. * $p < 0.05$; ** $p < 0.01$; $p < 0.001$ (see Methods for details).
